# Editorial: Applications of AI in autonomous, surveillance, and robotic systems

**DOI:** 10.3389/frobt.2025.1616634

**Published:** 2025-06-06

**Authors:** Patrick Sebastian

**Affiliations:** Electrical and Electronics Engineering Department, Faculty of Engineering, Universiti Teknologi PETRONAS, Seri Iskandar, Perak, Malaysia

**Keywords:** editorial, AI, robotics, surveillance, autonomous

## 1 Introduction

The rapid advancement of Artificial Intelligence (AI) has ushered in a new era of intelligent systems capable of autonomous decision-making, real-time perception, and adaptive learning. Across robotics, unmanned systems, and surveillance technologies, AI is transforming how machines interact with the world—enhancing precision, efficiency, and autonomy. This editorial provides an overview of key research contributions in this domain, highlighting the pivotal role of AI in shaping the future of autonomous navigation, robotic manipulation, and multi-agent surveillance systems.

The selected papers in this issue address critical challenges in visual perception, swarm coordination, and secure data management, demonstrating how AI-driven solutions are pushing the boundaries of what autonomous systems can achieve. From Visual SLAM in robotics to blockchain-secured UAV swarms, these studies showcase both the current state of the art and future directions for research and deployment.

## 2 AI in autonomous robotics: perception, navigation, and manipulation

### 2.1 Evolution of visual SLAM for robotics

The paper *“A Review of Visual SLAM for Robotics: Evolution, Properties, and Future Applications”* provides a comprehensive survey of how Simultaneous Localization and Mapping (SLAM) has evolved with deep learning and computer vision (Al-Tawil et al.). Traditional SLAM systems relied heavily on geometric algorithms, but modern AI-powered approaches leverage neural networks for feature extraction, dynamic object tracking, and semantic understanding. This shift enables robots to operate in complex, unstructured environments—from industrial warehouses to disaster zones. Future research directions include lightweight SLAM for edge devices and multi-agent collaborative mapping.

### 2.2 Task-aware robot navigation

In *“Image-based Robot Navigation with Task Achievability,”* the authors explore AI-driven navigation strategies that go beyond simple path planning (Ishihara1 et al.). By integrating reinforcement learning and vision-based perception, robots can assess environmental constraints and adjust their trajectories to ensure task completion. This is particularly valuable in logistics, where mobile robots must dynamically reroute around obstacles while maintaining operational efficiency.

### 2.3 Overcoming the reality gap in robotic grasping

A major challenge in robotic manipulation is the discrepancy between simulated training data and real-world execution. The study *“6IMPOSE: Bridging the Reality Gap in 6D Pose Estimation for Robotic Grasping”* introduces a novel AI framework that improves 6D pose estimation—critical for precise grasping in industrial automation and healthcare robotics (Cao et al.). By combining synthetic data augmentation with domain adaptation techniques, the proposed method significantly enhances robotic dexterity in unstructured settings.

## 3 AI in autonomous UAVs and multi-agent surveillance

### 3.1 Safe autonomous UAV docking

Unmanned Aerial Vehicles (UAVs) are increasingly used for surveillance, delivery, and infrastructure inspection, but their operational endurance is limited by battery life. The paper *“Vision-based Safe Autonomous UAV Docking with Panoramic Sensors”* presents an AI-powered docking system that allows UAVs to autonomously land on charging stations using panoramic vision (Nguyen et al.). This innovation enhances mission longevity while reducing human intervention, paving the way for fully autonomous drone fleets.

### 3.2 Cooperative Surveillance with heterogeneous swarms

The integration of UAVs, Unmanned Ground Vehicles (UGVs), and Unmanned Marine Vehicles (UMVs) into a cohesive surveillance network is explored in *“UAV-UGV-UMV Multi-Swarms for Cooperative Surveillance.”* AI-driven coordination enables these systems to share sensory data, optimize coverage, and adapt to dynamic threats (Stolfi1 et al.). Applications range from border security to environmental monitoring, where multi-domain swarms provide unparalleled situational awareness.

### 3.3 Blockchain for secure Multi-UAV surveillance

As UAV swarms become more prevalent, ensuring data integrity and preventing cyber threats is paramount. *“Towards a Blockchain-Based Multi-UAV Surveillance System”* proposes a decentralized AI framework where blockchain technology secures communication between drones (Santos De Campos et al.). This approach mitigates risks such as spoofing and data tampering, making autonomous surveillance systems more resilient in adversarial environments.

## 4 Future challenges and ethical considerations

While AI-powered autonomous systems offer immense potential, several challenges remain:

• Robustness in Real-World Conditions: AI models must generalize across diverse environments without excessive retraining.
• Human-AI Collaboration: Ensuring safe interaction between autonomous systems and human operators is crucial.
• Ethical and Privacy Concerns: Surveillance applications must balance security needs with individual privacy rights.
• Energy Efficiency: Prolonging operational endurance for UAVs and robots remains a key hurdle.

Future research should focus on explainable AI for autonomous decision-making, edge computing for real-time processing, and regulatory frameworks to govern AI deployment in sensitive applications.

## 5 Conclusion

The papers featured in this issue demonstrate the transformative impact of AI on autonomous, surveillance, and robotic systems. From enhancing robotic perception to enabling secure multi-agent coordination, these innovations are setting the stage for smarter, more resilient autonomous technologies. As the field progresses, interdisciplinary collaboration—spanning AI, robotics, cybersecurity, and ethics—will be essential to realizing the full potential of these systems while addressing societal concerns.

This editorial serves as a gateway to the cutting-edge research presented in this collection, inviting further exploration into how AI continues to redefine the boundaries of autonomy and intelligence in machines.

